# You can treat my HIV – But can you treat my blood pressure? Availability of integrated HIV and non-communicable disease care in northern Malawi

**DOI:** 10.4102/phcfm.v9i1.1151

**Published:** 2017-02-15

**Authors:** Colin Pfaff, Vera Scott, Risa Hoffman, Beatrice Mwagomba

**Affiliations:** 1 Partners in Hope Medical Center, Malawi; 2 School of Public Health, University of Western Cape, South Africa; 3 Department of Medicine and Division of Infectious Diseases, David Geffen School of Medicine at UCLA, United States; 4 University of St Andrews, Scotland, United Kingdom; 5 College of Medicine, University of Malawi, Blantyre, Malawi; 6 NCD Unit, Ministry of Health, Malawi

## Abstract

**Background:**

Many patients on antiretroviral therapy (ART) in Malawi have or will develop non-communicable diseases (NCDs). The current capacity of ART sites to provide care for NCDs is not known.

**Aim:**

This study aimed to assess the capacity of ART sites to provide care for hypertension and diabetes in rural Malawi.

**Setting:**

Twenty-five health centres and five hospitals in two rural districts in northern Malawi.

**Methods:**

A cross-sectional survey was performed between March and May 2014 at all facilities. Qualitative interviews were held with three NCD coordinators.

**Results:**

Treatment of hypertension and diabetes was predominantly hospital-based. Sixty percent of hospitals had at least one clinician and one nurse trained in NCD care, whereas 5% of health centres had a clinician and 8% had a nurse trained in NCD care. Hundred percent of hospitals and 92% of health centres had uninterrupted supply of hydrochlorothiazide in the previous 6 months, but only 40% of hospitals and no health centres had uninterrupted supply of metformin. Hundred percent of hospitals and 80% of health centres had at least one blood pressure machine, and 80% of hospitals and 32% of health centres had one glucometer. Screening for hypertension amongst ART patients was only conducted at one hospital and no health centres. At health centres, integrated NCD and ART care was more common, with 48% (12/25) providing ART and NCD treatment in the same consultation.

**Conclusions:**

The results reflect the status of the initial stages of the Malawi NCD programme at sites currently providing ART care.

## Introduction

Non-communicable diseases (NCDs) are the largest cause of mortality in the world accounting for 63% of the 57 million deaths globally.^1^ Of these deaths, 80% occur in low- and middle-income countries. In 2001, 20% of mortality in sub-Saharan Africa was estimated to be because of NCDs,^2^ and this is estimated to rise to 46% by 2030.^3^ In Malawi in 2011, 28% of all deaths were estimated to be because of NCDs^4^ with adult prevalence rates of 32.9% and 5.6% for hypertension and diabetes, respectively.^5^ Historically, NCDs have received little attention in Malawi; however, this has changed in the last 5 years. In 2011, Malawi included NCDs in the Essential Health Package^6^ and followed this with the development of a National NCD Action Plan.^7^ This plan focuses on cardiovascular disease, diabetes, chronic kidney disease, chronic respiratory disease, cancers, epilepsy, mental health and injuries. In the same year, a National NCD Unit at the Ministry of Health was established.

With the establishment of a highly successful anti-retroviral therapy (ART) programme in Malawi, HIV is now managed as a chronic disease. Increasingly calls are being made to recognise the overlap between HIV and NCD care as both being chronic diseases and to integrate care of the two conditions.^8^

Samb et al.^9^ undertook an extensive review of all published work in Cochrane and PubMed databases from 1999 to 2009 relating to health systems aspects of prevention, diagnosis, treatment, monitoring and management of chronic diseases in low-income and middle-income countries. As a framework, they used the WHO building blocks of health systems.^10^ They conclude that chronic disease programmes are very dependent on health systems that are well functioning and equitable and as such chronic disease programmes often expose weaknesses in the six building blocks of the health care system.

Indeed, the health systems in many African countries have weaknesses that will need to be addressed if NCDs are to be controlled effectively. Drugs to manage NCDs are either unavailable or unaffordable for patients.^11,12,13,14^ Equipment is often lacking.^15,16^ Health personnel are not trained adequately or are not present in sufficient numbers. Monitoring and evaluation systems often do not include NCDs, and use of health information is weak.^17^ Many health systems are orientated to acute episodic care, and significant changes will need to be made to adjust to the patient centred care that is needed for chronic disease control.^18^ All of these gaps will need to be identified and corrected if NCDs are to be managed effectively.

## Aim and objectives

This study aimed to examine the current capacity of the NCD programme, using hypertension and diabetes services as tracer conditions, in two rural districts in northern Malawi, and to explore the extent of integration with the HIV and ART care.

## Research methods and design

### Study design

This study used sequential mixed methods in the form of a cross-sectional facility survey and semi-structured interviews with key informants. The survey was based on a tool from the World Health Organization Package of Essential NCD interventions for Primary Health Care^19^ focusing on service delivery and referral systems, the availability of key resources (human resources, diagnostic equipment and medication) and programme management support (such as health information and programme supervision and planning). The semi-structured qualitative interviews were conducted with the NCD coordinators in both districts and at the central hospital. Questions were asked about the role of NCD coordinators and the strengths and weaknesses of the current NCD programme (Appendix 1).

### Setting

The study was performed under the umbrella of the Extending Quality Improvement for HIV/AIDS in Malawi (EQUIP) project - a USAID funded HIV programme aimed at improving care in hospitals and health centres in these two districts.

### Study population and sampling strategy

The study was conducted at all 25 health centres and five hospitals supported by the EQUIP project in two rural northern districts of Malawi. This excluded 13 health centres (eight in one district, five in other districts) not supported by EQUIP at the time of the study. The NCD coordinators were two clinical officers and one nurse. All had been appointed as coordinators between April 2012 and March 2013 and had been in their position for at least 12 months.

### Data collection

Data were collected between March and May 2014. Interviews were conducted by the same interviewer, who was the researcher and had previous experience in qualitative research.

### Data analysis

For the quantitative questionnaire, summary statistics were generated using Microsoft Excel 2010. The qualitative interviews were transcribed, and the texts were then reviewed to identify key themes deductively (informed by a review of the literature) and inductively (new themes emerging from the data collected). The themes suggested by the literature review before data collection were framed by the six WHO building blocks of health systems. A further two themes emerged inductively from the text after the data collection. The data from the qualitative interviews were triangulated with the quantitative data from the questionnaire to check validity of the findings.

## Ethical considerations

Permission for research was granted by the Research and Ethics committee of the University of Western Cape and the Malawi National Health Sciences Research Committee, and a non-human subjects designation was made by the University of California, Los Angeles, Internal Review Board.

## Results

### Service delivery and referral

Using hypertension and diabetic care as tracers of NCDs, this study found that there were three models of providing NCD care operating in the EQUIP-affiliated facilities (see Table 1): a stand-alone NCD clinic (two hospitals), NCD care as part of a specialist medical clinic (central hospital) and NCD patients treated as part of general outpatients department (OPD) (two hospitals and all health centres).

**TABLE 1:** Service delivery of care and screening services.

Facility type	Hospital 1: Mission hospital	Hospital 2: Mission hospital	Hospital 3: District hospital	Hospital 4: Rural hospital	Hospital 5: Central hospital	Health centres
**NCD service delivery**						
Model of NCD delivery	General OPD	Dedicated NCD clinic	Dedicated NCD clinic	General OPD	Specialist medical OPD	General services
Total OPD consultations per facility in February 2014^a^,^c^	2343	1062	7271	5870	9885	1850^c^
Consultations for hypertension per facility in February 2014^b^	56 (2.4%)	31 (2.9%)	234 (3.2%)	29 (4.9%)	593 (6%)	12 (0.7%)^c^
Consultations for diabetes per facility in February 2014^b^	10 (0.4%)	1 (0%)	55 (0.9%)	6 (0.1%)	238 (2.4%)	0 (0%)^c^
**Integration of NCD and ART care**						
Both in same consultation	N	N	N	N	N	48%^c^
Both same day, different consultation	N	Y	Y	Y	N	40%^c^
Both services available but on different days	Y	N	N	N	Y	12%^c^
**Screening for NCDs at OPD**						
Weight	Y	Y	N	N	N	0%^c^
Height	N	N	N	N	N	0%^c^
Blood pressure	Y	Y	N	N	Y	0%^c^
Glucose	N	N	N	N	N	0%^c^
**Screening for NCDs at ART clinic**						
Weight	Y	Y	Y	Y	Y	100%^c^
Height	Y	Y	Y	Y	Y	88%^c^
Blood pressure	Y	N	N	N	N	0%^c^
Glucose	N	N	N	N	N	0%^c^

*Source:* Cross-sectional quantitative survey

OPD, outpatients department; NCDs, non-communicable diseases; ART, Antiretroviral Therapy; Y, yes; N, no; NA, not applicable.

a All OPD consultations including NCD consultations.

b Percentages of total OPD consultations are shown in brackets under total or average numbers.

c Average for 25 health centres.

The central hospital OPD attended to more patients per month (9885) compared with two government hospital OPDs (7271 and 5870) and two mission hospital OPDs (2343 and 1062) (see Table 1). Relatively more patients were treated for hypertension at the central hospital (593 patients representing 6% of all OPD visits) compared with the two government hospitals (234 and 29 each, representing 4.9% and 3.2% of all OPD visits) and the two mission hospitals (56 and 31 each, representing 2.4% and 2.9% of all OPD visits). Approximately three-quarters of all diabetic patient visits took place at the central hospital (*n* = 238, 77%), with the government hospitals treating fewer diabetic patients (55 and 6 patients each) and the two mission hospitals also treating very few patients (10 and 1 patients each). Similarly, health centres treated very few patients with hypertension (average = 12) and none with diabetes in one month.

All facilities offered ART services: hospitals had been providing these services for a median of 104 months and health centres for 28 months. The degree of integration between ART and NCD services varied between hospitals and health centres (see Table 1). None of the hospitals provided ART and NCD treatment during the same consultation although this was provided at 48% (12/25) of the health centres. Patients could receive ART and NCD treatment on the same day at 60% (3/5) of hospitals and 40% (10/25) of health centres, but had to attend different consultations. Patients had to return on a different day to receive ART and NCD medication at 40% (2/5) of the hospitals and 12% (3/25) of health centres.

Two hospitals, both mission hospitals, stand out in performing routine screening of weight and blood pressure in OPD settings (see Table 1). In ART clinics, weight was measured at all facilities and height in all hospitals and 88% of health centres. However, routine screening of blood pressure in the ART clinic was only performed at one mission hospital. Blood pressure and weight were routinely measured on all patients at dedicated NCD clinics but glucose readings were only performed in the case of known diabetics or suspected new patients. There was no consistent pattern between screening for blood pressure and weight in OPD across the three models of care.

All facilities reported that they were able to refer patients for NCD care to a higher level, but 50% of the referring hospitals (2/4) and 28% (7/24) of the health centres reported that inadequate availability of ambulances was a major barrier to referral. Public transport was still the most frequent means to transport emergency patients at 40% of health centres. Eighty-four percent of health centres reported that it took less than one hour to move by car to the nearest referral facility and only 8% that it took more than 2 hours by car.

### Availability of key resources: equipment, guidelines, trained staff and medication

Most (88%) health centres had at least one clinician available who could prescribe NCD medication, whereas 80% had both a clinician and a nurse (see Table 2). In terms of workload, the hospitals saw an average of 56 OPD consultations per clinician per day with an average of 17 patients with hypertension and 4 patients with diabetes per clinician per day, although there was wide variation between hospitals. Health centres saw 66 patients per clinician per day, with insignificant numbers of patients with hypertension and diabetes. Training in NCD care was more established at hospitals compared with health centres: three hospitals reported that at least one clinician and one nurse had been trained in NCDs, whereas only one health centres (4.7%) reported any training for clinicians and only two (8.3%) reported training for nurses on NCDs.

**TABLE 2:** Availability of key resources, including trained staff, equipment and medication.

Variable	Hospitals (*n* = 5)			Health centres (HCs) (*n* = 25)		
	Average number functional	Average number not functional	Hospitals with working equipment (%)	Average number functional	Average number not functional	HCs with functional equipment (%)
**Availability of key equipment**						
BP machines (manual or automated)	2	1.8	100	1.6	2.1	80
Weighing scales	1.8	0.8	100	1.6	1	96
Height meter	1.6	0	100	1	0.2	92
Ophthalmoscope	0.2	0.8	20	0	0.2	4
Glucometer	2.6	0.2	80	0.4	0	32
Biochemistry analyser	0.4	0.2	40	0	0	0
**Availability of trained staff and guidelines**						
At least one clinician able to prescribe NCD medication	100%			88%		
Clinician workload^a^	56 OPD consultations per clinician per day (in addition to inpatient responsibilities)			66 patients per clinician per day		
At least one clinician trained	60%			4.7%		
At least one nurse trained	60%			8.3%		
Clinical Guidelines book on NCD management^a^	20%			72%		
**Availability of medication in past 6 months**						
Facilities with stock outs of tracer first-line HPT drug^b^	0%			8%		
Facilities with stock outs of tracer second-line HPT drug^c^	80%			96%		
Facilities with stock outs of tracer first-line DM drug^d^ (%)	60%			100%		
Facilities with stock outs of tracer second-line DM drug^e^	40%			100%		
Facilities with stock outs of tracer NCD suppliesf	60%			92%		

*Source*: Cross-sectional quantitative survey

BP, Blood pressure; NCDs, non-communicable diseases; NA, not applicable.

a Percentage of facilities that had a copy of ‘Malawi Standard Treatment Guidelines’ or ‘The Clinical Book’ present in the consulting room.

b Hydrochlorthiazide.

c Calcium channel blocker.

d Metformin.

e Insulin.

f Glucose test strips.

All of the hospitals and 80% of the health centres (20/25) had functional blood pressure machines (see Table 2). However, accuracy checks had either not been performed or were not known to have been performed at any of the hospitals and all but one of the health centres. Eighty percent of the hospitals and 32% of health centres had a working glucometer. Forty percent of hospitals were able to analyse biochemistry in the laboratory. Twenty percent of hospitals and 72% of health centres had copies of clinical guidelines on NCD management available in the clinic for use by clinicians.

Hydrochlorothiazide was the most widely available NCD drug, with all hospitals and 92% of health centres having uninterrupted supply in the past 6 months (see Table 2). Second-line anti-hypertensive medication was much less available with only 20% of hospitals and 4% of health centres reporting an uninterrupted supply of calcium channel blockers. Diabetic medication was also frequently interrupted at hospital level, with only 40% of hospitals having an uninterrupted supply of metformin and 60% of hospitals having an uninterrupted supply of insulin, whilst this was completely unavailable at health centres.

### Management support systems

Data on NCDs were recorded in the OPD register and admission and discharge registers at all hospitals and health centres. Information on hypertension, diabetes and other NCDs was otherwise not recorded in the Health Management Information System (HMIS) template that was submitted to the District Health Office (DHO) monthly. Although the National NCD had issued a template for quarterly reporting on NCDs, only two hospitals (40%) and two health centres (8%) submitted any quarterly NCD report directly to the NCD unit.

Only one health centre and none of the hospitals reported any NCD supervision visits from the district and only two hospitals but none of the health centres (6.7% of all facilities) reported any NCD supervision from the NCD unit at the Ministry of Health in the past 12 months.

In the qualitative interviews, all three NCD coordinators concurred that ongoing drug and equipment shortages had been a major challenge during the period they had been NCD coordinators.

‘We had sporadic supplies of glibenclamide – but at the moment we don’t have. It’s the thing stopping me from starting a clinic …. I’m looking for all drugs to be available so that we have a choice.’ (Participant 1, Male, Clinician)

Both district NCD coordinators reported that they were not able to visit health centres in the district because of lack of transport.

‘I have never visited the health centres. I came in 2011. We only had 2 routine DHO visits (to the health centres) but I was unable to participate in others because of a problem with transport. I have not been able to start again.’ (Participant 1, Male, Clinician)

They also reported many challenges in data collection. The advantage of a dedicated NCD clinic emerged as a desired service delivery model with respect to quality of care and patient education.

‘There is a lack of stationary – there are not enough report forms. There is a lack of paper and toner used to produce them. Also the photocopier broke down so we can’t photocopy the forms. I was using my money to copy the NCD reporting forms – at the copy bureaux.’ (Participant 1, Male, Clinician)

Two broader themes emerged in the qualitative data: how the coordinators saw their roles, and the sense of agency they felt in that role. NCD coordinators saw their main role as providing clinical care rather than supporting the initiation and development of NCD services in the health centres. The three coordinators seemed to display different degrees of agency regarding their ability to improve the system of NCD care in their districts. This variation was between coordinators and also between issues. Coordinators mentioned having some sense of agency in improving drug supply or coordinating laboratory tests, but seemed powerless to bring change in other areas, such as equipment, coordinating colleagues or doing community outreach.

‘So there are all these complaints. There is nowhere where they can bring these complaints so bring them to me. I liaise with these patients and the management. And all the clinicians. I say the patients are complaining this and this and this. I make sure that they change.’ (Participant 3, Male, Clinician)

## Discussion

### Service delivery and referral

One of the most striking findings revealed in this study is that the NCD care in the EQUIP-affiliated facilities in two rural northern Malawi districts was being primarily delivered at the central referral hospital, with district hospitals carrying a much smaller load and the health centres providing almost no NCD care. As 32% of the population is hypertensive and 5% have diabetes,^5^ there is a need to bring care as close to the patient as possible. Beaglehole et al.^20^ argue that primary care facilities are best positioned to prevent and manage NCDs. In Malawi, this will require a reversal in the current locus of care, which is predominantly at a secondary and tertiary level. The results from this study suggest a large current unmet need for diabetes and hypertension care. The combined population of the two districts is 678 965 people. The national STEPS survey^5^ indicates a hypertensive prevalence of 32% and diabetes prevalence of 5.6% in adults aged 25–65 years. Using a population estimate in the combined districts that adults in the age range 25–65 years make up 29.7% of the total population,^21^ there is an estimated hypertension burden of 64 528 people and diabetes burden of 11 293 people in these two districts. This research showed that in February 2014, only 943 people received treatment for hypertension and 310 for diabetes at the sites included in this study. This estimate suggests that only 1.5% of patients with hypertension and 2.7% of patients with diabetes received care in these two districts in this month.

A large community study in Malawi showed that 94.9% of adults with hypertension were unaware of their diagnosis and were not on treatment.^5^ Similarly, it is estimated that 78% of those with diabetes in sub-Saharan Africa remain undiagnosed.^15^ In this study, the lack of hypertension screening in adults at all OPD and health centres service delivery points represents missed opportunities for case detection. The potential to detect hypertension by screening at an ART clinic is demonstrated by one study in Malawi, where blood pressure measurement as part of a research study found that 45.9% of 174 patients on a long-term ART had raised blood pressure.^22^

### Availability of key resources: equipment, guidelines, trained staff and medication

Human resources have been listed as the major limitation for health services in Africa to develop.^23,24^ The high current workload in these two districts makes it clear that in order to adequately service the need for hypertension and diabetic care, a much greater workforce would be needed. Task shifting to other cadres, as used successfully in providing HIV care, has also been suggested as a strategy to manage NCDs in these same settings where human resources are limited.^20^ In particular, there is evidence to show that nurses can achieve good outcomes in hypertension and diabetes clinics in South Africa, Cameroon and Ethiopia.^24,25^ However in Malawi, most NCD care is also already provided by non-physician clinicians and nurses, and the potential to further task shift to other cadres is limited. More staff will be needed if the burden of NCD is to be met.

This study has revealed a shortage of equipment to manage hypertension and diabetes at all levels of the health service. Other studies in Africa have revealed similar challenges. The rapid assessment survey conducted in Mozambique found that only the minority of facilities had glucometers.^11^ In rural South Africa, 90% of the blood pressure machines at rural primary care clinics had faulty valve function and only one cuff met the size recommendations of the South African Hypertension Society guidelines.^16^ Clearly, more equipment will need to be provided if NCDs are to be diagnosed and managed effectively in Malawi.

Hospitals did not have a consistent supply of second-line anti-hypertensives and anti-diabetic drugs. Similar circumstances have been described elsewhere in Africa. In Mozambique, insulin was poorly available in hospitals and not available at all in health centres.^11^ In Swaziland, the availability of drugs to manage diabetes was quite variable.^15^ In Ethiopia, the availability of anti-hypertensive drugs was variable.^12^ In urban and rural Tanzania and Cameroon, it was found that hypertension was the only condition treated consistently at primary health care level, whilst diabetes was largely treated at secondary and tertiary care.^14^ The large study of six low- and middle-income countries including Malawi found that less than 7.5% of drugs were available at all sites, although there was better availability at private facilities than public facilities.^26^ In sum, these findings support the broad challenge in drug supply faced by many African countries and raise a need to better understand the challenges of the supply chain to make improvements that will ultimately translate into successful NCD programmes.

### Management support systems

A reliable health information system is the cornerstone of managing a health system; it enables drug forecasting, planning, decision making and resource allocation.^27^ NCDs, although part of the Essential Health Package of Malawi, are not routinely recorded as part of the HMIS. This has been found in many other countries where NCDs are not part of routine health information systems.^9,28^ Although donor funding has led to vastly improved data collection for HIV, this has often not strengthened the overall health information systems.^9^ Several authors point out that the focussed monitoring and evaluation as used in the ART programme in Malawi could easily be applied to NCDs.^27,28^

### Potential for integration

Integration of NCD care with other health services may be an advantage for several reasons. In particular, there has been much discussion in the literature about the potential and synergies in integrating NCD care with ART care. Both Wagner’s Chronic Care Model^29^ and WHO’s Innovative Care for Chronic Conditions^30^ have been recommended as the ideal model for chronic care services and both would apply to NCD care and ART, making synergies between the two programmes obvious. Both diseases require adherence to chronic medication, blood tests to monitor for complications and lifestyle modification.

Integration has been a strong focus in the implementation of ART services, where ART has been integrated into PMTCT, TB, child health and reproductive health services. In general, different models of this integration have been implemented, ranging from a referral model to a co-location model to a one-stop-shop fully integrated model.

Very few countries have tried to fully integrate NCD and ART services to date, and indeed, different aspects of integration may be relevant or applicable in different contexts. In Ethiopia, it was decided that complete integration of clinical services was not the best way forward, but an intermediate approach was developed where systems were shared between programmes such as guidelines, training, drug procurement laboratory and monitoring and evaluation.^15^ In Rwanda, a mental health and epilepsy programme had been developed by the late 1990s and a well functioning ART programme by 2005. As these programmes were already functioning well, it was decided to pilot an integrated NCD programme in which various NCDs were managed together but not to integrate with these other stand-alone services.^31^

In this study, all NCD coordinators strongly supported the establishment of separate NCD clinics as opposed to having patients seen in OPD. They saw the advantage of a separate clinic being in terms of providing separate patient education, including patients educating each other, ensuring vital signs are taken on all patients and having clinician time more focussed on providing specific care issues. However, interviewees were much more hesitant about integrating ART care with NCD care, citing stigma and work load as the main concern, although they did admit that integration with ART services would be more convenient to patients with comorbidities.

### Strengths and limitations

There were several limitations in the methodology of this study. In completing the cross-sectional survey, the most senior person on duty that day was interviewed, and in some cases, this was not the person in charge of the facility. The health centres were purposefully selected as sites where the EQUIP project was already working. As these sites had been receiving support for ART services, they may be better resourced than others in the district. As these two districts have lower population densities and are less urban than other regions in Malawi, the findings may not be generalisable to other districts in Malawi. However, they reveal important health system challenges in rural Malawi. Only three qualitative interviews were conducted, and these only represented the views of the NCD coordinators. It would be useful to reflect the views of clients and communities in further research.

### Conclusions

The Malawi NCD programme has made important first steps in its development. However, many gaps remain in drug delivery, human resource training, health information, supervision and leadership. Currently, a very small minority of patients with NCDs are being treated, predominantly within hospital settings rather than in health centres. Given the current workload observed, increased staff are needed to meet the demand for NCD screening and care. There is much potential to use the lessons from the ART programme to strengthen NCD care as ART clinics represent one of the first screening and primary care delivery models for chronic disease in Africa, supported by health information, short course training and supervision.

## Appendix 1

### Interview guide

#### Guide for semi-structured interview for focal persons for NCDs in each district and at central hospital.

1. What are your responsibilities as focal person for NCD?
2. In what way do you feel supported in doing your job?

What tools/guidelines/report formats/meetings/lines of communication do you have to assist you in this responsibility?

In what way would you like to be better supported and resourced?

3. What relationship do you have to the health facilities in your district? Describe, giving some examples.
4. What training/policy updates have happened or are planned in your district around NCD care? (Probe for target audience, frequency and mentorship/support post training)
5a. In your view, what are the current strengths of delivery of NCD care in the **district** and why?
5b. In your view, what are the main weaknesses of delivery of NCD care the **district**?
6a. In your view, what is current strengths of delivery of NCD care in the **central hospital** and why?
6b. In your view, what are the main weaknesses of delivery of NCD care in the **central hospital**?
7. Describe the referral system for patients with NCD. What are the strengths and weaknesses of this system?
